# Effects of Cardiovascular, Resistance and Combined Exercise Training on Cardiovascular, Performance and Blood Redox Parameters in Coronary Artery Disease Patients: An 8-Month Training-Detraining Randomized Intervention

**DOI:** 10.3390/antiox10030409

**Published:** 2021-03-09

**Authors:** Tryfonas Tofas, Ioannis G. Fatouros, Dimitrios Draganidis, Chariklia K. Deli, Athanasios Chatzinikolaou, Charalambos Tziortzis, George Panayiotou, Yiannis Koutedakis, Athanasios Z. Jamurtas

**Affiliations:** 1 School of Physical Education and Sport Science, University of Thessaly, 42100 Trikala, Greece; tr_tofas@pe.uth.gr (T.T.); ifatouros@uth.gr (I.G.F.); ddraganidis@pe.uth.gr (D.D.); delihar@pe.uth.gr (C.K.D.); y.koutedakis@uth.gr (Y.K.); 2 School of Physical Education and Sport Science, Democritus University of Thrace, 69100 Komotini, Greece; achatzin@phyed.duth.gr; 3 Department of Health Sciences, European University Cyprus 6 Diogenis Str., 2404 Engomi, P.O. Box 22006, 1516, Nicosia, Cyprus; c.tziortzis@euc.ac.cy (C.T.); G.Panayiotou@euc.ac.cy (G.P.); 4 School of Sports, Performing Arts and Leisure, University of Wolverhampton, Walsall Campus, Gorway Rd, Walsall WS1 3BD, UK

**Keywords:** cardiovascular disease, oxidative stress, aerobic, health, blood pressure, antioxidants

## Abstract

It is well-documented that chronic/regular exercise improves the cardiovascular function, decreases oxidative stress and enhances the antioxidant capacity in coronary artery disease (CAD) patients. However, there is insufficient evidence regarding the chronic effects of different types of training and detraining on cardiovascular function and the levels of oxidative stress and antioxidant status in these patients. Therefore, the present study aimed at investigating the effects of cardiovascular, resistance and combined exercise training followed by a three-month detraining period, on cardiovascular function, physical performance and blood redox status parameters in CAD patients. Sixty coronary artery disease patients were randomly assigned to either a cardiovascular training (CVT, *N* = 15), resistance training (RT, *N* = 11), combined cardiovascular and resistance training (CT, *N* = 16) or a control (C, *N* = 15) group. The training groups participated in an 8-month supervised training program (training three days/week) followed by a 3-month detraining period, while the control group participated only in measurements. Body composition, blood pressure, performance-related variables (aerobic capacity (VO_2max_), muscle strength, flexibility) and blood redox status-related parameters (thiobarbituric acid reactive substances (TBARS), total antioxidant capacity (TAC), reduced glutathione (GSH), oxidized glutathione (GSSG), catalase activity (CAT), protein carbonyls (PC)) were assessed at the beginning of the study, after 4 and 8 months of training as well as following 1, 2 and 3 months of detraining (DT). CVT induced the most remarkable and pronounced alterations in blood pressure (~9% reduction in systolic blood pressure and ~5% in diastolic blood pressure) and redox status since it had a positive effect on all redox-related variables (ranging from 16 to 137%). RT and CT training affected positively some of the assessed (TAC, CAT and PC) redox-related variables. Performance-related variables retained the positive response of the training, whereas most of the redox status parameters, for all training groups, restored near to the pre-exercise values at the end of the DT period. These results indicate that exercise training has a significant effect on redox status of CAD. Three months of detraining is enough to abolish the exercise-induced beneficial effects on redox status, indicating that for a better antioxidant status, exercise must be a lifetime commitment.

## 1. Introduction

Over the years, different studies have reported that coronary artery disease (CAD) and endothelial dysfunction are characterized by chronic inflammation and oxidative stress [1,2]. In addition, several studies have indicated that oxidative stress plays an important role in the pathogenesis and development of CAD including atherosclerosis, ischemia-reperfusion injury, chronic ischemic heart disease, cardiomyopathy, heart failure, hypertension, dyslipidemia, diabetes mellitus, myocardial infraction, angina pectoris and ensuing arrhythmias [3,4]. However, there is strong evidence that mild, repeated exercise-induced oxidative stress and the related adaptations up-regulate endogenous antioxidant defense mechanisms [5], which might paradoxically improve health and longevity in CAD patients [6]. Furthermore, healthy tissue responds favorably to oxidative stress after chronic exercise by increasing endogenous antioxidant system activity to maintain redox homeostasis [7], in contrast with non-healthy or aged muscles [7,8].

It is widely known that chronic exercise reduces oxidative stress and damage, by both decreasing reactive oxygen species (ROS) production and increasing antioxidant capacity as well as improving mitochondria efficiency in several organs and systems [9]. In addition, the exercise-induced oxidative stress might itself be beneficial, by reducing arterial antioxidant enzymes in different tissues: heart, liver, blood or muscle. Recent evidence suggests that both aerobic and anaerobic exercise training are beneficial to improve redox balance in humans, and any type of exercise training will be beneficial for improving the redox balance against potential risk factors of excessive ROS-mediated diseases [10].

Although several studies confirm the benefits of regular physical exercise on redox status, it has also been shown that an acute physical exercise with a certain high intensity and duration may induce an increase in the production of ROS [11,12]. However, a repeated bout of exercise attenuated muscle damage and blood oxidative stress compared to the first bout [13]. Thus, regular exercise training seems to be an efficient way of reducing the susceptibility of muscles to exercise-induced damage and several studies have suggested that this protection is associated with an increased activity of muscle antioxidant enzymes, including superoxide dismutase, catalase and glutathione peroxidase as well as antioxidants such as vitamin C, vitamin E, carotenoids and glutathione [14,15].

It is likely that aerobic fitness is related to higher antioxidant capacity. In addition, cardiovascular training (CVT) can trigger exercise-associated adaptive responses through metabolic and redox challenges [16,17]. The decrease in oxidative damage associated with exercise training could also be explained by an increase in antioxidant and metabolic efficiency, which possibly prevents the stimulation of DNA repair enzyme activity [18]. These findings enhance the importance of regular exercise in the prevention of DNA damage accumulation, which has been related to aging [19] and some age-related diseases including cardiovascular disease [20]. Furthermore, both chronic aerobic exercise [16,21] and resistance exercise [22] improve muscle mitochondrial density, as well as decrease oxidative stress in different tissues [23].

The long-lasting effects of exercise on oxidative stress and its relationship with cardiovascular diseases is controversial, mainly because of the differences among the type, intensity, frequency and duration of the exercise programs found in the literature. Moreover, most of the studies concerned with physical exercise and related oxidative stress have concentrated on the effects of CVT [24,25], resistance training (RT) [26] or a combined cardiovascular and resistance training (CT) [18,27,28] on oxidative stress mainly in healthy individuals.

In contrast to exercise training redox adaptations, there is limited data regarding the detraining effects on oxidative stress markers [2]. Most detraining studies have focused on the effects on muscular strength [29,30,31,32,33], lipid metabolism [34,35,36,37], body composition [37,38] bone mineral density [39], functional fitness [40], memory function [41] and cardiovascular response [42,43,44,45]. Nevertheless, it still remains unclear whether training adaptations persist [29,32,46,47,48] or whether they are completely lost [40,42,44,45] after a detraining period (DP).

To the best of our knowledge, no previous study has evaluated the effect of exercise training followed by a DP on redox status in CAD patients. The few studies that investigated the detraining effects on oxidative stress were conducted either on rats [2,43,49,50] or healthy individuals [2], and the training period was too small. In addition, no information exists on whether different training modes (CVT, RT and CT) have accordingly different effects on redox status markers after a detraining period. Therefore, the present study aimed at comparing the effects of cardiovascular, resistance and combined exercise training, followed by a three-month detraining period, on cardiovascular function, physical performance and blood redox status in CAD patients.

## 2. Methods

### 2.1. Participants and Experimental Design

The main goal of the present study was to assess the efficacy of CVT, RT and CT on modulating the redox status as well as the impact of a subsequent detraining period in CAD patients. Recruitment focused on low-risk patients according to the criteria established by the American Association of Cardiovascular and Pulmonary Rehabilitation [51].

**Figure 1:**
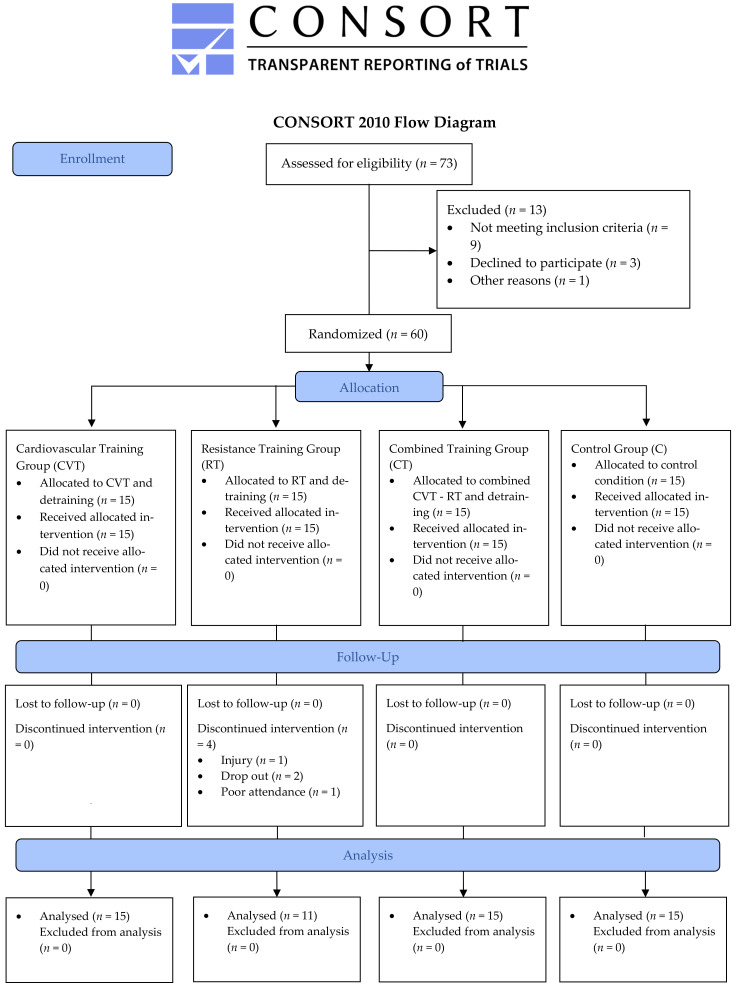
CONSORT 2010 flow diagram.

A preliminary power analysis using the G*Power software (3.0.10) revealed that a sample of 8–12 participants per group was required to detect statistically meaningful differences between four groups with six repeated measurement points (effect size >0.55, α error probability of 0.05, two-tailed α level power of 0.9). Patients with CAD were initially recruited through information given to health care providers, posting, newspaper, media advertisements and by word of mouth. All prospective subjects completed a health history questionnaire, were examined by a physician and underwent resting and exercise standard echocardiography and ECG. They were included in the study if they: (a) were low-risk CAD patients, (b) did not display angina or other significant symptoms (e.g., unusual shortness of breath, light headedness or dizziness), (c) did not display pathologic ECG-changes at rest or during exercise stress test, (d) were not characterized by technical limitations such as poor echocardiographic image quality, (e) were free of uncontrolled congestive heart failure, uncontrolled diabetes mellitus, unstable dysrhythmia and uncontrolled systemic hypertension, (f) were free of arthritis or other musculoskeletal and inflammatory diseases, (g) were not either currently or previously using anti-inflammatory drugs or tobacco products and (h) they were free of any musculoskeletal injury. Seventy-three individuals were initially recruited and examined for eligibility. All participants had previously undergone coronary artery bypass grafting (CABG, *n* = 37) or percutaneous transluminal coronary angioplasty (PTCA, *n* = 36). From the initially recruited subjects, 60 subjects entered the study and 56 completed the study [two subjects withdrew from the study due to personal reasons, one due to muscle injury and another one due to poor attendance (<80% participation in training sessions)]. From the final entered subjects in the study, 15 of them had single-vessel disease, 28 had double-vessel disease, 11 had triple-vessel disease and 2 had quadruple-vessel disease. All subjects were receiving anticoagulant therapy. Figure 1 illustrates the CONSORT flow diagram.

**Figure 2:**
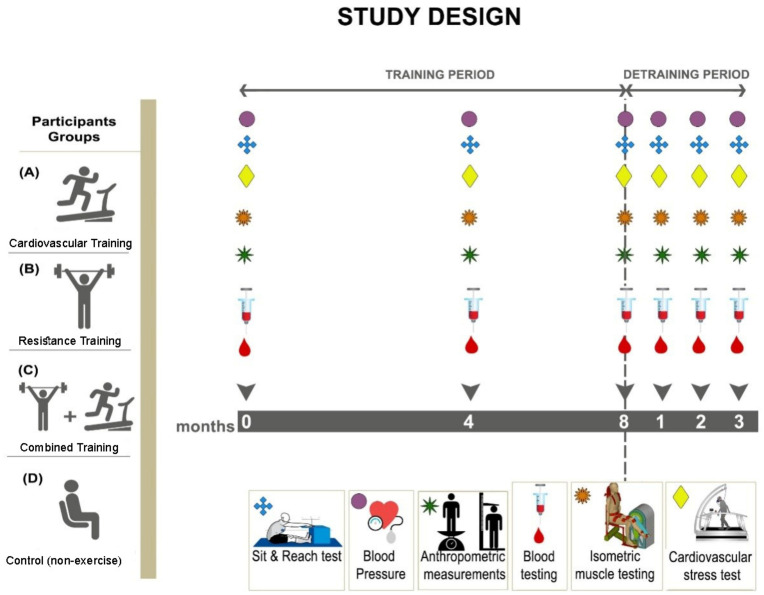
Study design and time points of data collection. (**A**), represents the Cardiovascular training group (CVT); (**B**), represents the Resistance training group (RT); (**C**), represents the Combined training group (CT); (**D**), represents the control group (C).

A controlled, randomized, four-group, repeated measures design was applied. Figure 2 illustrates the study design and the time points of data collection.

Participants were randomly assigned to: (i) a control group that participated only in measurements (C, N = 15, age = 64 ± 8 years, body mass = 86.0 ± 3.6 kg, height = 1.68 ± 1.4 m), (ii) a cardiovascular training group (CVT, *N* = 15, age = 61 ± 7 years, body mass = 87.5 ± 2.9 kg, height = 1.68 ± 1.4 m), (iii) a resistance training group (RT, *N* = 11, age = 62 ± 8 years, body mass = 88.7 ± 3.6 kg, height = 1.68 ± 2.6 m) or (iv) a combined training group (CT, *N* = 15, age = 64 ± 6 years, body mass = 85.2 ± 2.1 kg, height = 1.69 ± 1.7 m). All of the initially recruited patients had previously undergone coronary artery bypass grafting (CABG, *n* = 37) or percutaneous transluminal coronary angioplasty (PTCA, *n* = 36). Nineteen of them had single-vessel disease, 34 had double-vessel disease, 13 had triple-vessel disease, 5 had quadruple-vessel disease and 2 had quintuple-vessel disease. CABG and PTCA patients that completed the study were seven and eight, respectively, in the CVT group, four and seven, respectively, in the RT groups, eight and seven, respectively, in the CT group and seven and eight, respectively, in the C group.

Each of the three training groups (CVT, RT and CT) followed a supervised training program during the first eight months and subsequently abstained from training for three months (detraining period; months 9 to 11). Body composition (waist and hip circumference), systolic and diastolic blood pressure as well as blood sampling and performance measurements [Maximal oxygen consumption (VO_2max_), muscle strength and flexibility] were performed at baseline, after 4 and 8 months of training and monthly during detraining.

All participants were fully informed about the aim of the study and the experimental procedures, as well as the associated risks and benefits, before obtaining written consent. Procedures were approved by the Cyprus National Bioethics Committee (Code#: ΕΕΒΚ/ΕΠ/2006/37) and were completed in accordance with the declaration of Helsinki.

### 2.2. Training Intervention

All training protocols were performed under supervision, three times per week, on non-consecutive days, over an 8-month period. Each session started at the same time of the day (to avoid circadian variations) with a 10-min warm up including low-intensity cycling or running on a treadmill and stretching, and ended with a 5-min cool-down. Participants were advised to maintain their normal dietary habits throughout the training period and the subsequent 3-month detraining phase. Exercise training took place in a university exercise gym that was fully equipped to support the training programs of the participants.

Cardiovascular training (CVT): CVT was conducted on a treadmill or a cycle ergometer and consisted of 10-min intervals repeated four times at 60–75% HR_max_, interspersed by 6 min recovery periods. Both running and cycling speed was individually adjusted, to ensure that all subjects exercised at their prescribed exercise intensity, and was based on a graded exercise testing performed at baseline and after 4 months of training.

Resistance training (RT): The RT program comprised of eight different exercises (chest press, shoulder press, pulley row, total abdominal, rotary torso, leg press, leg extension, leg curl) performed in circuit fashion, targeting muscle groups of the upper and lower body. Each session lasted 50–60 min and included two sets of 12–15 repetitions per exercise, with 60–90 sec rest between exercises and a 5 min resting period between sets. The exercise intensity was set at 60% of the one-repetition maximum (1RM). The 1RM was determined for each exercise at baseline and after 4 months of training to maintain the desired workload throughout the 8-month training period.

Combined training (CT): The CT program incorporated CVT and RT during the same session, with each session lasting 50–60 min. After warm up, participants performed two 10-min intervals at 60–75% of HR_max_ on a treadmill or a cycle ergometer, interspersed by 6-min recovery periods. Then, they proceeded with RT, during which they executed one set of 12–15 repetitions in the same exercises as in RT, with a 60–90 sec rest between exercises at an intensity corresponding to 60% of 1RM.

Control group (C): Participants in C group did not undergo any formalized physical training. They were asked to maintaining their current habitual physical activity levels and participated only in testing procedures.

### 2.3. Anthropometric Measurements

Body mass and height were measured to the nearest 0.5 kg and 0.5 cm, respectively, using a beam balance with stadiometer (Beam Balance-Stadiometer, SECA, Vogel & Halke, Hamburg, Germany) as described [52]. Waist and hip circumferences were measured according to the World Health Organization’s data gathering protocol (WHO, 2012). The waist circumference was assessed at the midpoint between the lower margin of the last palpable rib and the top of the iliac crest, using a stretch-resistant tape that provides a constant 100 g tension. Hip circumference was measured around the widest portion of the buttocks, with the tape parallel to the floor.

### 2.4. Resting Blood Pressure Assessment

Systolic and diastolic blood pressure were measured according to the standardized procedure developed by the American Heart Association [53]. The measurement performed using a calibrated, manual sphygmomanometer with the participant in the supine position and after having rested for 5 min. Systolic and diastolic pressures were recorded using the first and fifth Korotkoff sounds, and each assessment was performed in duplicate, with the two values being averaged.

### 2.5. Performance Testing

Flexibility of the lower back and hamstring muscles was assessed following a 5-min warm up by using the modified sit-and-reach test, as described [54]. Maximal isometric peak torque of knee extensors (IPTE) of the right leg was measured using a computer-controlled isokinetic dynamometer (Cybex Norm Lumex, Ronkonkoma, NY, USA), as previously described [55]. Prior to testing and following an 8-min warm up on a cycle ergometer (Excite, Technogym, Italy), a familiarization protocol with submaximal (< 50% max) isometric repetitions on the isokinetic dynamometer was applied. During isometric testing, three maximal repetitions were performed (3-sec duration) at 60° of knee joint flexion, with a 60 sec rest interval in-between, and the maximal torque value (N^.^m) was recorded. Visual feedback and verbal encouragement were continuously given during assessment [56]. The test-retest reliability of the IPTE was 0.97.

### 2.6. Cardiovascular Stress Testing

The cardiovascular stress testing was performed according to the Bruce protocol (Bruce RA and Hornsten, 1969) using a graded multistage treadmill and Ultima™ CardiO_2_ gas exchange analysis system (St. Paul, Minnesota, USA) for the determination of maximal oxygen uptake (VO_2max_), as described [57]. Initially, treadmill speed was set at 2.7 km/h and gradient was adjusted at 10%. Speed and gradient were incrementally increased after each 3-min stage by ~1.5 km/h and 2%, respectively, and the test was ended when the subject reached their maximal exercise capacity (within 6 to 12 min).

Twelve lead electrocardiogram (ECG), heart rate and blood pressure measurements were performed during each 3-min stage of the test as well as over a 5-min period post-testing. Patients were continuously encouraged to reach their maximum exercise capacity and the test was terminated when subjects indicated exhaustion or the ECG revealed an abnormal rhythm or ischemia. Moreover, the onset of chest pain, significant ST segment depression, arrhythmia or non-cardiac symptoms during the exercise led to premature termination of the test.

VO_2max_ was determined when three of the following four criteria were met: (i) volitional fatigue, (ii) a < 2 mL/kg/min increase in VO_2_ following an increase in work rate, (iii) a respiratory exchange ratio ≥ 1.10 and (iv) heart rate equal or greater than 85% of the subject’s predicted HR_max_ (calculated as 220 age). Peak oxygen consumption (VO_2peak_) was determined as the highest 20-s average value of VO_2_ observed during the last 60 s of exercise. Gas analyzer calibration was performed prior to each subject’s testing.

### 2.7. Blood Sampling and Assays

All blood samples were obtained between 08:00–09:00 (to avoid circadian variations) after an overnight fast. Samples (~10 mL) were drawn from an antecubital arm vein using a 20-gauge disposable needle equipped with a Vacutainer tube holder (Becton Dickinson, Franklin Lakes, NJ, USA) with the subject in a seated position. For serum separation, a blood portion (~4 mL) was collected into a Vacutainer tube, allowed to clot for 30 min at room temperature and subsequently centrifuged (1500× *g*, 4 °C, 15 min). The supernatant was dispensed in multiple aliquots (into separate microcentrifuge Eppendorf™ tubes) and stored at −80 °C for later analyses of total antioxidant capacity (TAC). Another blood portion (~4 mL) was collected into a Vacutainer tube containing ethylenediaminetetraacetic acid (EDTA) and centrifuges immediately (1370× *g*, 4 °C, 10 min) for plasma separation. The supernatant (plasma) was collected into microcentrifuge Eppendorf™ tubes (multiple aliquots) and stored at −80 °C for later analyses of thiobarbituric acid reactive substances (TBARS) and protein carbonyls (PC). Erythrocytes into the Vacutainer tube were lysed as described [58], and the lysate was dispensed in multiple aliquots and stored at −80 °C for later analysis of catalase (CAT), reduced glutathione (GSH) and oxidized glutathione (GSSG).

Analysis of TAC, TBARS, CAT, GSH, GSSG and PC were performed according to protocols that have been described previously [59,60]. Briefly, for TAC analysis, serum samples were mixed with sodium-potassium phosphate (10 mM, pH 7.4) and 2.2-diphenyl-l picrylhydrazyl (0.1 mM), incubated in the dark, at room temperature over a 30-min period, immediately after being centrifuged (20,000× *g*, for 3 min) and had their absorbance read at 520 nm. TBARS were assayed by adding 35% TCA (200 mM) and Tris-HCL (pH 7.4) to plasma samples that were mixed and incubated at room temperature for 10 min. Then, Na2SO4 (2M) and thiobarbituric acid (55 mM) were added to the solution, and incubated at 95 °C for 45 min. After incubation, samples were allowed to cool for 5 min, had 70% TCA added, mixed and centrifuged (15,000× *g*, for 3min) and the absorbance of the supernatant was then measured at 530 nm. CAT activity was determined in red blood cell (RBC) lysate. Initially, sodium-potassium phosphate (67 mM, pH 7.4) was added to samples, mixed and then incubated at 37 °C for 10 min. Subsequently, 30% hydrogen peroxide was added and the change in absorbance was read at 240 nm over 90 s. For GSH analysis, RBC lysates were mixed with sodium-potassium phosphate (67 mM, pH 8.0) and 5.5-dithiobis-2-nitrobenzoate (1 mM), following treatment with 5% TCA, and then incubated in the dark at room temperature for 45 min. After incubation, their absorbance was read at 412 nm. For GSSG measurement, samples (RBC lysates) were initially treated with 5% TCA (pH 7.0–7.5). Then, 2-vinyl pyridine was added and samples incubated at room temperature for 2 h. After incubation, samples were mixed with sodium phosphate (143 mM, pH 7.5), NADPH (3 mM), 5.5-dithiobis-2-nitrobenzoate (10 mM) and distilled water and incubated at room temperature for 10 min. Thereafter, glutathione reductase was added and samples’ absorbance was read at 412 nm over 3 min. Hemoglobin in red blood cell lysate was determined using a commercially available kit (Dutch Diagnostics BV, Zutphen, The Netherlands), in order to estimate the final levels of GSH, GSSG and catalase. For the determination of hemoglobin, 10 µl of erythrocyte lysate treated with 5% TCA were mixed with 2500 µl of working reagent (pH 7.3; diluted 1:10). The samples were immediately vortexed and left for at least 3 min at 25 °C. For the determination of PC in plasma, samples were mixed with 20% TCA, incubated in an ice bath for 15 min and centrifuged (15,000× *g*, 4 °C, 5 min). Then, the supernatant was discarded and 2.4-dinitrophenylhydrazine (10 mM in 2.5 N HCL) was added to the samples, while HCL (2.5 N) was added to each blank. Thereafter, samples and blank solutions incubated in the dark at room temperature for 1 h with transient mixing every 15 min. After incubation, samples and blank solutions centrifuged (15,000× *g*, 4 °C, 5 min), 10% TCA was added to the pellet (after discarding the supernatant) and centrifuged again at 15,000 g, 4 °C for 5 min. The supernatant was then removed, ethanol-ethyl acetate (1:1 *v*/*v*) was added to the pellet and centrifuged for 5 min (15,000× *g*, 4 °C). The last procedure repeated two more times, and thereafter, the supernatant was discarded, the pellet mixed with 5 M urea (pH 2.3) and incubated at 37 °C for 15 min. Finally, the samples and blank solutions centrifuged for 3 min (15,000× *g*, 4 °C) and the absorbance of the supernatant read at 375 nm. All spectrophotometric assays were performed by using a Hitachi 2001 UV/VIS (Hitachi Instruments Inc., Tokyo, Japan) and inter- and intra-assay coefficients of variations in all assays ranged from 2.4 to 7.5% and from 3.4 to 8.1%, respectively.

### 2.8. Statistical Analysis

Data are presented as means ± SD and all statistical analyses were performed using the IBM SPSS software (IBM SPSS Statistics 20). A 2X6 repeated measures ANOVA was used to identify group and time differences and possible interactions. One-way repeated measures ANOVA was used to identify time-related differences separately for each group. Normality was verified using the Shapiro–Wilk test. The level of statistical significance was set at *p* < 0.05.

## 3. Results

No sustained arrhythmias or other cardiovascular complications were observed in any of the patients during exercise. There were no differences between the four groups of participants in relation to the baseline characteristics in any of the assessed variables.

### 3.1. Anthropometric, Physiologic and Performance Variables

Hip circumference (Table 1) was significantly lower after 4 months of training in all training groups and the control group and was significantly lower after 3 months of detraining in CVT and RT groups as well as in the C group. Waist circumference was significantly lower after 4 months of training in CVT and RT groups and the C group and was also significantly lower after 3 months of detraining in CVT and RT groups as well as in the C group. However, the waist to hip ratio did not change significantly at any time point of assessment.

Blood pressure responses appear on Table 2. After 4 months of exercise training, systolic blood pressure (SBP) was significantly reduced in the CVT and CT groups group and remained reduced until the end of the third month of the DP. SBP decreased significantly after 8 months of exercise training and was restored back to the baseline values at the end of the first month of the DP. SBP values in the CVT, RT and CT groups were significantly different compared to the C group values after 8 months of training. Diastolic blood pressure (DBP) decreased significantly after 4 months only in the CVT group and it remained significantly lower compared to baseline until the end of the 2nd month of the DP. DBP decreased significantly after 8 months of resistance training but was restored back to the initial value after one month of detraining. DBP values for the CVT and RT groups at 8 months of training was significantly lower compared to the control group.

**Figure 3:**
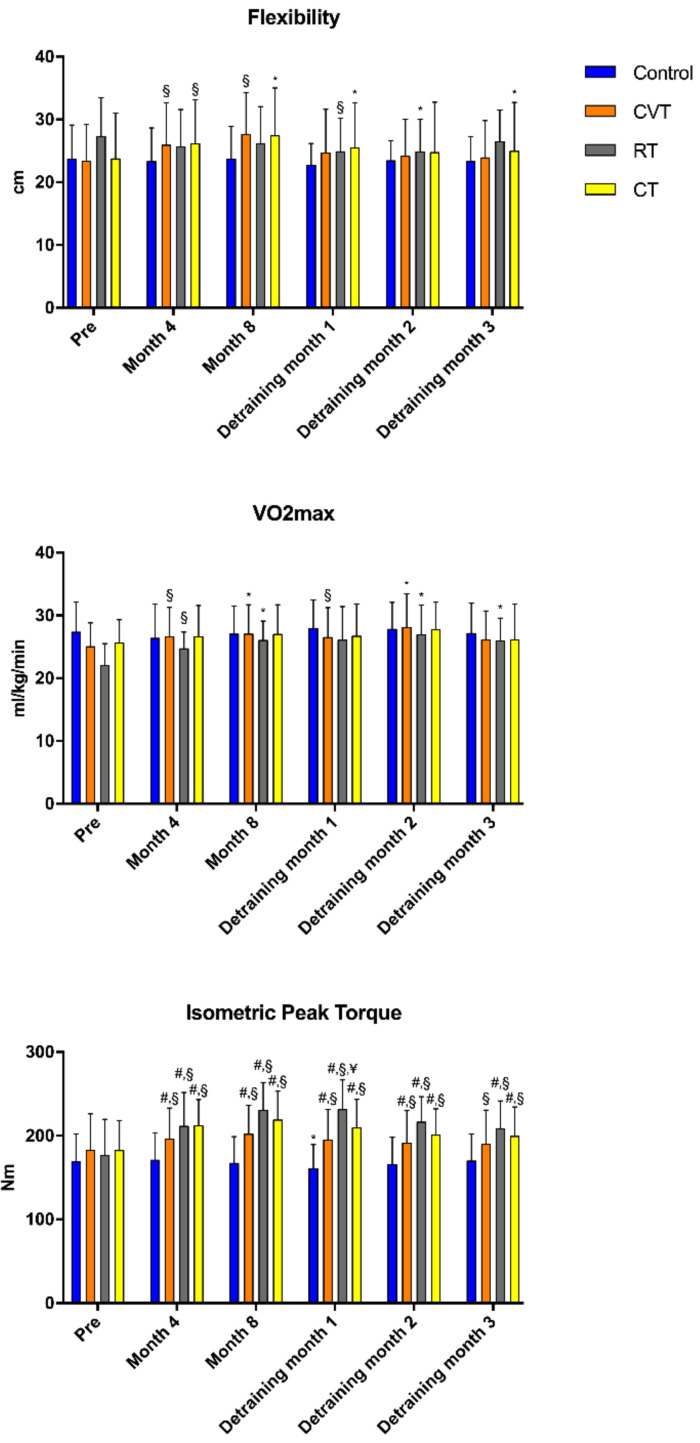
Performance-related responses following training and detraining in cardiac patients. *Sig vs. pre in the same group (*p* < 0.05), ^§^ Sig vs. pre in the same group (*p* < 0.001), ^#^ Sig vs. Control in the same time point (*p* < 0.05), ^¥^ Sig vs. CVT in the same time point, CVT: Cardiovascular training, RT: Resistance training, CT: Combined training.

Performance-related responses following training and detraining appear on Figure 3. Flexibility increased significantly after 4 months of CVT and CT training and remained elevated at 8 months of training. The CT group retained the flexibility increases until 3 months after the end of training. Flexibility was significantly lower one and two months into detraining in the RT group. Aerobic capacity (VO_2max_) was significantly increased at 4 (11%) and 8 months (18.5%) following the CVT and remained significantly elevated for two months into DP. RT resulted also in elevated aerobic capacity at 4 (12%) and 8 (18%) months of training, a response that remained significantly increased at 3 months of DP. No significant changes were observed in the CT following the training period. Training resulted in significant improvement in IPTE in all training groups, and this response was retained during the DP. Furthermore, IPTE values of the training groups were significantly higher compared to the control group following the training and after the DP.

### 3.2. Redox Status Variables

There were no significant differences between groups at pre-exercise levels for the assessed redox status variables. CVT resulted in significant elevations of GSH at 4 (40%) and 8 (45%) months of training (Table 3). However, one month of detraining abolished the aforementioned elevations. The other two forms of training did not result in any change of GSH. GSSG was significantly reduced by approximately 20% following CVT and this was the only form of training that resulted in significant changes of this variable. The lower levels of GSSG were sustained until the first month of detraining before reverting to the initial levels. The previous responses of GSH and GSSG resulted in a significant increase by 210% and 180% after 4 and 8 months of training, respectively. However, the ratio of GSH to GSSG returned to baseline values within a month of detraining. All forms of exercise training resulted in reduced levels of PC and TBARS at 4 months of training. However, PC returned to baseline levels at 8 months of training and remained at this level during DP. TAC responses were significantly greater following all forms of training with TAC levels returning to baseline levels during detraining, with RT being the exception, since it retained a positive response at 3 months of DP.

## 4. Discussion

To the best of our knowledge, this is the first study that investigated the effects of three different types of exercise on cardiovascular, performance and redox status markers, in CAD patients, for 8 months of exercise training and 3 months of detraining. It seems that the CVT has influenced the most performance and redox parameters compared to the other types of exercise. In our study, patients in the CVT group improved their body composition, reduced their blood pressure, increased their flexibility, muscle strength and VO_2max_ and affected positively and over a longer period of time most of the redox parameters that were assessed.

### 4.1. Cardiovascular and Physical Characteristic Parameters

Even though the exercise training resulted in significant changes in waist and hip circumferences, the waist to hip ratio was not altered in any group of assessment. Previous work has shown that compared to baseline, 8 months of training resulted in body mass reduction ranging from 2.5% to 4.5% with body mass remaining lower into detraining in the CVT and CT groups, whereas it returned to baseline values in the RT group [34]. Assessing the waist circumference and hip circumferences separately, it was found that both measurements were mainly reduced in CVT and CT groups, but the ratio was not significantly altered in any group. It is well known in the literature that the energy expenditure achieved through exercise along with possible negative energy balance through exercise can lead to changes in body composition. Reduction of waist circumference and circulating triglycerides along with improvement in cardiorespiratory fitness has been proposed as a measure to improve cardiovascular health and decrease the cardiometabolic risk [61].

Taking into consideration the negative consequences of hypertension, non-pharmaceutical strategies need to be developed to combat this condition. Lifestyle modifications that promote healthy living with proper nutrition and exercise are factors that have been proposed as means to decrease blood pressure. Exercise training resulted in significant changes in SBP and DBP, with profound changes appearing following the CVT and CT, and these results were maintained throughout the detraining period. The percent decrease of SBP due to training ranged from 8.5 to 18.6%, whereas the decrease in DBP was approximately 5%. Previous research has shown similar results with this study. Aerobic exercise training resulted in a significant decrease of SBP and DBP by approximately 10 and 7%, respectively [62,63], whereas a recent systematic review and metanalysis indicated that exercise interventions elicit significant reductions in blood pressure, with aerobic exercise showing the most significant reductions in 24-h, daytime and nighttime ambulatory blood pressure [64]. The blood pressure results of our study could be attributed to several factors including reduced peripheral vascular resistance, sympathetic nervous system [65] as well as favorable changes in inflammatory status, endothelial function, arterial compliance and oxidative stress [66]. Furthermore, several parameters of the redox status were improved following exercise training in this study and the reduced oxidative stress and inflammation could in part explain the positive effects of exercise training on blood pressure. Taken together, these results suggest that in an intervention exercise training program for CAD patients, the type of exercise that has shown to be the most important is aerobic exercise.

Performance (flexibility, VO_2max_, IPTE) was increased following exercise training and the different types of training produced different time-dependent changes. CVT and combined training resulted in significant changes in flexibility that remained elevated even after 3 months of detraining. On the other hand, RE training did not produce any significant change, and not only that, it decreased during the 3 months of detraining assessment. These results are in contrast with previous work which indicates that resistance training by itself improves flexibility in the aged [67]. However, in the aforementioned study, it was reported that flexibility changes were intensity-dependent and intensities greater than 60% of 1RM were more effective in producing flexibility gains. The intensity of our resistance training protocol was at 60% of 1RM, and thus the intensity may be was not high enough to improve flexibility. Aerobic performance was increased only after CVT and resistance training and remained elevated for almost all the detraining period. Previous research has shown similar results with this study as far as exercise training is concerned [68]. It was surprising not to see any significant changes in aerobic performance following the combined training. Perhaps the stimulus of the aerobic and resistance training itself in the combined training was not high enough to induce significant changes in aerobic capacity. IPTE was increased after exercise training regardless of the type of training. RT produced the greatest absolute increases and these gains remained significantly higher into DP. These results are in accordance with previous studies, which have shown that muscular strength of elderly individuals, when subjected to resistance training of moderate to high intensity, can be maintained above baseline levels during 2 to 31 weeks of detraining [29,30,31,32,33]. However, the novel finding of this study is that aerobic capacity and strength gains of low-risk cardiac patients are remained even during the DP. It is, therefore, important to understand that the benefits of exercise training can be maintained for a prolonged period of time following the cessation of a training program and also to pinpoint that additional research is needed to clarify the maximum time of maintenance of these positive effects.

### 4.2. Redox Status

It is known that cardiovascular diseases and endothelial dysfunction are related to chronic inflammation and oxidative stress [1,69] and increased levels of ROS contribute to vascular dysfunction, both in animal models and clinical studies [70,71,72,73]. ROS at low levels might have a beneficial effect in vascular tone and endothelial and cardiac function. However, when there is an overwhelming ROS production, it can disturb the cellular function and result in cell damage [3,4]. There are several studies that indicate that regular exercise can up-regulate the expression of major antioxidant enzymes and reduce the pro-oxidant molecules [74,75]. Furthermore, the exercise-mediated generation of free radicals seems to be essential for the activation of cellular transduction pathways that will promote beneficial adaptations leading to an antiatherogenic effect [9]. The results from this study that examined the effects of exercise training on redox status support the aforementioned findings. Most of the variables assessed were positively affected after training with CVT showing the most significant time-dependent changes. Results from research involving animals and humans are similar with the ones of this study, which have shown that CVT training improve antioxidant capacity [25,74,76,77,78]. Healthy older individuals that exercised for 24 weeks with a moderate intensity for 3 days per week showed lower lipid peroxidation and inflammation and higher total antioxidant activity [25]. A study with a similar design to this one in which untrained men participated and exercised for 8 weeks revealed that all three training groups (CVT, resistance, combined training) elevated antioxidant capacity and had lower lipid peroxidation [28]. Furthermore, obese older individuals that followed a resistance training program improved their strength and aerobic capacity and decreased the levels of lipid peroxidation [26]. In addition, low intensity aerobic exercise training prevented the decline of antioxidant activity that is linked with aging [79]. Finally, Soares et al. [18] showed that oxidative damage to DNA decreased while physical fitness and total antioxidant capacity increased in healthy men following 16 weeks of combined CVT and RE training. Taken collectively, the results from our study indicate, for the first time, that exercise training in low-risk cardiac patients may increase the antioxidant potential and decrease markers of oxidative stress. This is of great importance since improvement in redox status will attenuate the risk of a recurrent cardiac episode.

### 4.3. Detraining

The term ”detraining” refers to complete or partial loss of training-induced adaptations, in response to absence of or insufficient training stimulus [80]. Notably, it has been proposed that the effect of detraining on CVDs and oxidative stress is equally important with that of exercise training, as it regulates the time-frame during which the cardiovascular-related adaptations will be diminished [45]. Since unpredicted reasons can result in a short or longer temporary break of training, it is of great importance to know which type/mode of exercise will affect mostly the detraining effects. Our study showed that CVT was the type of training that resulted in the most prominent effects that were retained into DP. Previous work on this subject is limited. However, a recent study suggested that moderate-intensity physical exercise of sufficient duration leads to beneficial adaptations on redox state of rats, which may be partially lost during detraining period [49]. Furthermore, Agarwal and colleagues suggest that 2 weeks of detraining is not long enough to completely abolish the exercise-induced beneficial effects [50]. In addition, Padilha and colleagues suggest that detraining for 12 weeks, does not completely reverse the changes on oxidative stress biomarkers in older women, induced by 12 weeks RT program [2]. On the contrary, previous studies reported that 3–4 months of detraining may lead to complete reversal of the cardio-protection offered by exercise training [81,82]. To our knowledge, there are no previous research on exercise training and detraining on cardiac patients. Previous work on the training-detraining effect on lipid profile in cardiac patients revealed that the CVT and the combination between CVT and resistance training had the most pronounced results on the lipid profile and inflammation [34]. Further studies are needed to elucidate the time-dependent changes of redox status following a detraining period.

## 5. Conclusions

Numerous studies have demonstrated that exercise training has a favorable effect on cardiovascular disease outcome. Results from this study indicate that systematic exercise training for 8 months results in reduction in blood pressure, increased exercise performance and favorable changes in redox status. These results are evident from the first 4 months of training and the blood pressure and performance responses can be retained for several months following cessation of training. However, most of the positive effects of redox status are abolished after 3 months of detraining. It seems that the aerobic exercise training is the form of training that has more notable and positive effects on redox status. Future research should assess different intensities and duration of exercise in an aim to identify the optimal exercise stimulus to provoke beneficial cardiovascular health in cardiac patients. Finally, a combination of exercise training with diet and/or supplement manipulations should also be assessed.

## Figures and Tables

**Table 1:** Waist and hip circumferences and their ratio following training and detraining in cardiac patients.

				Training						Detraining								
	Pre			Month 4			Month 8			Month 1			Month 2			Month 3		
*WAIST* (cm)																		
Control	98.5 ± 13.2			96.2 ± 12.5 *			97.6 ± 14.9			96.2 ± 13.7 *			95.1 ± 14.8 *			95.4 ± 14.8 *		
CVT	104.1 ± 7.7			102.1 ± 8.5 *			102.8 ± 8.4			102.7 ± 8.9 *			101.8 ± 8.6 *			101.7 ± 7.1 *		
RT	103.4 ± 10.8			102.3 ± 11.2			102.4 ± 11.2			102.2 ± 11.5			102.1 ± 11.3			101.4 ± 11.3		
CT	98.5 ± 6.9			96.9 ± 7.6 *			97.6 ± 7.7			96.9 ± 7.6			96.6 ± 7.04 *			96.2 ± 7.4 *		
*HIP (cm)*																		
Control	95.0 ± 9.3			93.6 ± 9.5 *			94.8 ± 10.8			93.5 ± 10.3 *			92.8 ± 9.4 *			92.0 ±10.5 *		
CVT	99.6 ± 7.9			98.2 ± 7.7 ^$^			99.3 ± 7.9			98.8 ± 8.9			97.4 ± 8.2 *			97.3 ± 7.7 *		
RT	100.7 ± 8.2			98.5 ± 8.6 *			99.6 ± 9.8			100.0 ± 8.7			98.9 ± 8.9			98.6 ± 9.2		
CT	95.5 ± 7.01			93.7 ± 7.5 *			95.3 ± 8.2			95.3 ± 7.4			94.3 ± 7.2			93.8 ± 7.0 *		
*WAIST/HIP*																		
Control	1.04	±	0.05	1.03	±	0.05	1.03	±	0.05	1.03	±	0.05	1.03	±	0.07	1.03	±	0.06
CVT	1.05	±	0.03	1.04	±	0.04	1.03	±	0.03	1.04	±	0.04	1.05	±	0.03	1.05	±	0.03
RT	1.02	±	0.03	1.04	±	0.04	1.03	±	0.05	1.02	±	0.05	1.03	±	0.04	1.03	±	0.03
CT	1.03	±	0.03	1.03	±	0.03	1.02	±	0.04	1.02	±	0.04	1.02	±	0.04	1.03	±	0.04

* Sig vs. pre in the same group (*p* < 0.05); ^$^ *p* = 0.05, CVT: Cardiovascular training, RT: Resistance training, CT: Combined training.

**Table 2:** Systolic and diastolic blood pressure responses following training and detraining in cardiac patients.

				Training						Detraining								
	Pre			Month 4			Month 8			Month 1			Month 2			Month 3		
SBP (mmHg)																		
Control	136.67 ± 4.21			135.33 ± 3.89			137.33 ± 4.39			136.33 ± 4.72			133.67 ± 5.09			132.33 ± 2.86		
CVT	140.28 ± 5.07			127.22 ± 3.92 *			128.33 ± 4.13 ^*,#^			127.22 ± 5.12 ^*,#^			129.17 ± 5.40 *			130.83 ± 4.07 *		
Resistance	138.64 ± 7.21			130.91 ± 4.11			122.73 ± 3.42 ^*,#^			126.82 ± 3.48 ^#^			130.91 ± 4.05			134.55 ± 5.02		
Combined	151.56 ± 7.64			128.75 ± 4.65 *			123.44 ± 3.20^*,#^			129.06 ± 3.12 ^*^			128.44 ± 3.32 *			129.06 ± 3.91*		
DBP (mmHg)																		
Control	81.67 ± 6.98			82.33 ± 4.95			83.33 ± 5.23			81.67 ± 5.56			81.33 ± 5.16			81.67 ± 5.87		
CVT	83.67 ± 8.12			79.66 ± 5.16*			78.8 ± 5.57 ^*,#^			79.33 ± 7.76 *			79.00 ± 4.70 *			80.33 ± 1.29		
Resistance	81.36 ± 7.10			81.82 ± 4.62			78.18 ± 8.14 ^$,#^			78.64 ± 3.23			78.64 ± 3.93			79.55 ± 4.71		
Combined	83.00 ± 8.16			80.66 ± 5.81			80.33 ± 5.81			81.00 ± 4.70			81.33 ± 2.28			81.33 ± 2.96		

* Sig vs. pre in the same group (*p* < 0.05), # Sig vs. Control in the same time point, ^$^ *p* = 0.06, SBP: Systolic Blood Pressure, DBP: Diastolic Blood Pressure, CVT: Cardiovascular training, RT: Resistance training, CT: Combined training.

**Table 3:** Redox status-related responses following training and detraining in cardiac patients.

	Training			Detraining		
	Pre	Month 4	Month 8	Month 1	Month 2	Month 3
*GSH (μmol/g Hb)*						
Control	4.13 ± 3.60	4.10 ± 1.445	3.23 ± 1.59	3.31 ± 1.19	3.61 ± 1.11	3.29 ± 1.36
CVT	3.74 ± 1.23	5.22 ± 2.13 *	5.45 ± 2.89 *	3.95 ± 1.92	3.33 ± 1.79	3.58 ± 2.80
RT	3.96 ± 2.21	4.98 ± 1.73	4.06 ± 2.55	2.96 ± 1.20	2.83 ± 1.03	3.43 ± 2.62
CT	4.21 ± 1.91	4.62 ± 2.38	4.18 ± 2.27	3.56 ± 1.57	3.96 ± 1.48	3.42 ± 1.98
*GSSG (μmol/g Hb)*						
Control	1.24 ± 0.38	1.17 ± 0.56	1.18 ± 0.64	1.17 ± 0.66	1.22 ± 0.43	1.26 ± 0.38
CVT	1.29 ± 0.31	1.03 ± 0.31 **^$^**	1.04 ± 0.37 **^$^**	1.06 ± 0.23 *	1.18 ± 0.20	1.35 ± 0.35
RT	1.10 ± 0.25	1.14 ± 0.27	1.08 ±0.38	1.01 ± 0.33	1.18 ± 0.31	1.31 ± 0.41
CT	1.18 ±0.37	1.05 ± 0.58	1.07 ± 0.57	1.00 ± 0.43	1.27 ± 0.31	1.32 ± 0.27
*GSH/GSSG*						
Control	3.40 ± 2.05	3.47 ± 1.60	3.28 ± 1.54	3.71 ± 2.36	3.46 ± 2.00	2.36 ± 0.85
CVT	2.91 ± 0.86	6.13 ± 6.09 *	5.30 ± 2.39 *	3.34 ± 1.66	2.88 ± 1.72	2.69 ± 2.09
RT	3.83 ± 2.39	4.75 ±2.27	3.49 ± 2.50	2.46 ± 1.38	2.31 ± 1.00	2.59 ± 1.66
CT	4.00 ± 1.87	5.04 ± 2.61	4.48 ± 3.09	3.70 ± 1.43	3.31 ± 1.49	2.52 ± 1.56
*Protein Carbonyl* *(nmol/g protein)*						
Control	0.73 ± 0.16	0.67 ± 0.34	0.76 ± 0.29	0.73 ± 0.17	0.73 ± 0.31	0.67 ± 0.17
CVT	0.66 ± 0.12	0.51 ± 0.13 *	0.68 ± 0.18	0.70 ± 0.14	0.72 ± 0.20	0.64 ± 0.17
RT	0.81 ± 0.18	0.41 ± 0.18 *	0.74 ± 0.11	0.83 ± 0.19	0.90 ± 0.15	0.70 ± 0.27
CT	0.82 ± 0.19	0.54 ± 0.13 *	0.80 ± 0.23	0.85 ± 0.18	0.90 ± 0.18	0.75 ± 0.22
*TBARS (μmol/L)*						
Control	5.65 ± 2.00	5.12 ± 2.80	5.73 ± 3.16	6.87 ± 2.80	6.86 ± 1.85	6.24 ± 3.04
CVT	5.95 ± 2.27	4.43 ± 2.45 *	5.97 ± 2.45	6.39 ± 2.36	6.28 ± 1.84	6.70 ±3.03
RT	6.16 ± 1.48	4.41 ± 2.33	5.98 ± 1.55	7.48 ± 1.51 *	6.78 ± 2.24	6.70 ± 2.27
CT	6.47 ± 3.43	4.77 ± 2.25	6.27 ± 1.78	7.36 ± 2.29	6.08 ± 1.50	7.47 ± 3.95
*TAC* (μmol DPPH/mL)						
Control	1.10 ± 0.15	1.14 ± 0.08	1.13 ± 0.07	1.08 ± 0.17	1.06 ± 0.12	1.03 ± 0.12
CVT	1.00 ± 0.13	1.15 ± 0.11 *	1.15 ± 0.08 *	1.00 ± 0.13	1.03 ± 0.10	1.03 ± 0.10
RT	0.93 ± 0.12	1.15 ± 0.09 *	1.12 ± 0.07 *	0.94 ± 0.13	1.01 ± 0.05 *	1.02 ± 0.05 *
CT	1.00 ± 0.10	1.17 ± 0.13 *	1.16 ± 0.12 *	1.03 ± 0.85	1.04 ± 0.10	1.05 ± 0.12
*Catalase (U/mg Hb)*						
Control	18.4 ± 7.5	23.3 ± 10.0	17.8 ± 12.6	20.0 ± 17.0	25.1 ± 6.5	17.7 ± 9.1
CVT	18.1 ± 18.4	43.0 ± 22.2 *	22.6 ± 15.4 **^$^**	20.7 ± 10.3	24.3 ± 12.3	16.5 ± 5.1
RT	13.8 ± 8.9	40.9 ± 22.5 *	23.1 ± 12.8 *	18.1 ± 5.9 **‡**	26.2 ± 12.9 *	18.5 ± 9.0
CT	14.9 ± 7.2	34.9 ± 14.8 *	29.5 ± 30.2 **‡**	21.3 ± 10.4 **‡**	22.2 ± 10.2 *	17.4 ± 7.0

* Sig vs. pre in the same group (*p* < 0.05), ^$^ Sig vs. pre in the same group (*p* = 0.05), ^‡^ Sig vs. pre in the same group (*p* = 0.07), CVT: Cardiovascular training, RT: Resistance training, CT: Combined training.

## Data Availability

All Data is contained within the article.
